# Phenolic Compounds, Vitamins C and E and Antioxidant Activity of Edible Honeysuckle Berries (*Lonicera caerulea* L. var. *kamtschatica* Pojark) in Relation to Their Origin

**DOI:** 10.3390/antiox11020433

**Published:** 2022-02-21

**Authors:** Jana Orsavová, Irena Sytařová, Jiří Mlček, Ladislava Mišurcová

**Affiliations:** 1 Language Center, Tomas Bata University in Zlín, Štefánikova 5670, 760 01 Zlín, Czech Republic; 2 Department of Food Analysis and Chemistry, Tomas Bata University in Zlín, Vavrečkova 275, 760 01 Zlín, Czech Republic; irena.hlavacova@email.cz (I.S.); mlcek@utb.cz (J.M.); l.misurcova@email.cz (L.M.)

**Keywords:** *Lonicera*, phenolics, chlorogenic acid, vitamin C and E, antioxidant activity, HPLC-DAD, Pearson correlation coefficient

## Abstract

Honeysuckles are frost tolerant plants providing early-ripening fruits with health-promoting properties which have been used in traditional medicine in China. This study evaluates the impact of the climatic conditions of two areas on the chemical composition and antioxidant activity (AOA; by DPPH—2,2-diphenyl-1-picrylhydrazyl and photochemiluminescence assays) of eight cultivars of honeysuckle berries (*Lonicera caerulea* L. var. *kamtschatica* Pojark) of various ripening times. Expectedly, chemical composition and AOA values varied depending on the cultivars, locality and selected methods. Berries from Lednice (the area with more sunshine) showed higher average contents of total monomeric anthocyanins (TMAC; pH differential absorbance method), vitamins C and E and total phenolics (high-performance liquid chromatography). In contrast, berries from Žabčice (the area with more rain) performed higher average contents of total phenolics and flavonoids (UV/VIS spectroscopic analyses). Interestingly, fundamental amounts of chlorogenic acid were determined irrespective of the locality. Regarding TMAC and vitamin C content, early ripening Amphora from both areas has been assessed as the best cultivar; concerning the content of phenolic compounds, Fialka from both areas and Amphora from Lednice is considered as the most valuable. The obtained results may facilitate the selection of the most valuable cultivars for both producers and consumers.

## 1. Introduction

Honeysuckle berries are edible fruits of the genus *Lonicera* from the *Caprifoliaceae* family counting approximately 180 species occurring mostly in the moderate zone of the northern hemisphere. The *Lonicera* shrub is extraordinarily frost-resistant and provides delicious early-ripening fruits known as honeysuckle berries or haskap [1]. As they are substantially rich in bioactive compounds, many cultivars with different ripening times, growing conditions and tastes are grown commercially in some European countries, including the Czech Republic, Poland, Slovakia, Lithuania, and Romania, as well as in Russia, Japan, and Canada [2].

Due to such considerable contents of bioactive compounds enhancing antioxidant potential, honeysuckle berries and extracts from leaves, flowers and branches possess many beneficial health properties, including antioxidant, anticancer, antibacterial, antiviral, antiseptic, anti-inflammatory, and anti-allergic [3]. Therefore, honeysuckle products are widely applied in traditional Chinese medicine and they are considered as a promising material for diverse pharmacological and cosmetics applications, the food industry and agriculture [4,5].

Generally, polyphenols are formed as secondary metabolites protecting plants from ultraviolet radiation, oxidants and pathogens and they reduce oxidative stress, one of the most significant factors in the progression of many chronic diseases. Their amount in plant bodies varies depending on several environmental and genetic factors. Among others, there are studies describing mechanisms of anti-tumor role of epigallocatechin-gallate (EGCG), quercetin (QUE), resveratrol (RES) and chlorogenic acid (CHL) targeting epigenetic mechanisms in breast, prostate, pancreatic, colon, lung and skin cancer [6]. In honeysuckle berries, anthocyanins and CHL comprise the majority of phenolic compounds [7]. Cyanidin-3-glucoside, CHL and catechins of honeysuckle berries are reported as a potential herbal agent to cure type 2 diabetes via the inhibition of α-amylase activity and reduction of postprandial hyperglycemia [8,9]. On the other hand, phenolic compounds may act as pro-oxidants if ingested in high doses [10].

Furthermore, honeysuckle berries are a rich source of vitamin C, an essential antioxidant molecule playing an important role in human metabolism as a cofactor of many enzymes involved in important processes, including the epigenetic control on gene expression [11].

Antioxidant activity (AOA) attributed to phenolic compounds and vitamins has been thoroughly studied as an important health-benefiting feature of plant raw material. Therefore, the impact of this potential of phenolic compounds in plant matter should be considered when assessing the bioactive value of food nutraceuticals [12]. However, AOA determined based on the chemical composition of fruits is also substantially affected by the genotype, time of harvest, environmental conditions [1], and by various mechanisms including synergic or antagonistic effects of the mixture of bioactive compounds presented in the plant matter [13,14], as well as their interaction with certain proteins that, via the regulation of gene expression, can eventually upregulate the cell’s endogenous antioxidant capacity [15]. Therefore, to obtain objective data, the application of diverse methods to determine AOA is recommended [16].

Generally, the content of bioactive compounds in fruits and vegetables seems to be strongly affected by abiotic and biotic factors, such as the pre-harvest climatic conditions (the light and temperature, their antioxidant role in the cellular system, quantity of nitrogen fertilizers, fruit maturity), harvest time, post-harvest procedures, storage time and genotypic differences and their antioxidant role in the cellular system [11,17]. Thus, changeable composition of bioactive compounds of honeysuckle berries, leaves and flowers has been documented in connection with the environmental conditions, locality, horticultural management, cultivars and ripening times, as well as applied extraction methods [7].

Indeed, it is necessary to investigate a broader group of cultivars to assess the variability of the composition of bioactive compounds in connection with various environmental conditions of different cultivation areas. That would facilitate a better selection of cultivars with a high nutritional value. Hence, this study examines the impact of diverse climatic and soil conditions of two different areas on the chemical composition of honeysuckle berries. It evaluates differences in berries of eight cultivars with the sequential ripening times, namely Altaj, Morena, Amphora, Fialka, Leningradskij velikan, Kamchadalka, Remont and Maistar, and also differences in the same cultivars grown in various areas. Spectrophotometric methods were used to determine total contents of phenolics (TP) by the Folin-Ciocalteu method, flavonoids (TF) by NaNO_2_, AlCl_3_·6H_2_O and NaOH and monomeric anthocyanins content (TMAC) by pH differential assay. Vitamins C and E and individual phenolic compounds were established using high-performance liquid chromatography with diode-array detector (HPLC-DAD) assays. Antioxidant activities (AOA) were established using free radical scavenging (DPPH) and photochemiluminescence (PCL) assays to cover different mechanisms of antioxidant effects of various compounds performing synergic or antagonistic effects. The links between AOA and individual phenolic compounds and vitamins C and E of honeysuckle berries were established by Pearson correlation coefficients (*r*) in the extent which has not been published yet. Based on the obtained results, the study identifies the most valuable cultivars from both investigated areas.

## 2. Materials and Methods

### 2.1. Fruit Samples

The study analyses honeysuckle berries of the following cultivars of *Lonicera caerulea* L. var. *kamtschatica* Pojark with various ripening times and origins: early ripening cultivars—Altaj (Slovakia), Morena and Amphora (Russia); medium early ripening cultivars—Fialka, Leningradskij velikan, Kamchadalka (Russia), and Remont (Czech Republic) and medium late-ripening cultivar—Maistar (Switzerland). Considering the genotypes of applied cultivars, the following genotypes are accessible: Altaj (*Lonicera kamtschatica* x *Lonicera turczaninowii*), Morena (*Lonicera kamtschatica* No. 101 x *Lonicera kamtschatica* No. 21-5 from Primorskii district), Amphora and Fialka (derived from free pollination of cultivar Roksana which is derived from free pollination of *Lonicera kamtschatica* from Tomskii district), Leningradskij velikan (seedlings of the third generation of *Lonicera kamtschatica* from Petropavlovsk district in Kamchatka), Kamchadalka (a variety of seedlings of *Lonicera kamtschatica* No. 15/63 from Primorskii district) and Remont (derived from seedlings obtained by free pollination of *Lonicera kamtschatica*). Berries of each honeysuckle cultivar were harvested from a minimum of five plants in the amount of 500 g per cultivar in a fully ripe state during May (early ripening), June (medium early ripening) and July (medium late ripening) in 2014 from two experimental areas of Mendel University in Brno. Fresh berries were homogenized by blender (Bosch MSM67170, Bosh GmbH, Stuttgart, Germany) and deep-frozen and stored in an Ultra-Low Temperature freezer (ULUF P610 GG—Arctiko, Esbjerk, Denmark) at −80 °C for at least 24 h and subsequently lyophilized by Alpha 1-4 LSC (Christ Gefriertocknungsanlagen GmbH Osterode am Harz, Germany) at −55 °C and 0.120 Mbar for 48 h. Lyophilized samples were homogenized by blender. The obtained powders were stored in zipper polyethylene bags at −20 °C until being analyzed.

### 2.2. Characteristics of Experimental Areas

**Figure 1:**
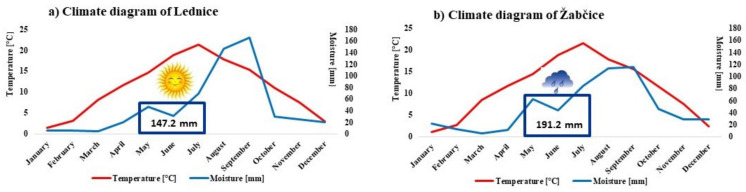
Climate diagrams of the experimental areas of Lednice (**a**) and Žabčice (**b**) in 2014 with highlighted differences in the sum of precipitation during the maturing season from May to July.

The experimental areas of Mendel University in Brno are located in the cadastral area of Lednice (177 m a.s.l., GPS coordinates: 48.7954925N, 16.7987622E;) and Žabčice (185 m a.s.l., GPS coordinates: 49.011598 N, 16.602572E). Both areas have typical continental climatic conditions with the same long-term average annual temperature of 9.2 °C and the precipitation of 479.7 mm (Lednice) and 519.0 mm (Žabčice). In 2014, the average annual temperatures in Lednice and Žabčice were 11.1 °C and 11.2 °C, respectively, and the precipitation reached 572.4 mm and 576.7 mm, respectively. During the maturing season from May to July 2014, the average temperature was almost the same in both localities—18.2 °C (Lednice) and 18.3 °C (Žabčice); however, the precipitation differed considerably: 147.2 mm in Lednice contrasting to 191.2 mm in Žabčice (Figure 1). Annual temperatures and sums of precipitation in 2014 and long-term values from both localities are shown in Table A1.

The area of Lednice is characterized by black, loamy-sandy soil with alkaline pH of 7.1 and a humus content of 1.56%. Žabčice is characterized by brown, glee alluvial soil with clayey-loamy topsoil with neutral or slightly acidic pH of 6.9 and a humus content of 2.28%.

### 2.3. Chemical and Reagents

Ethanol, methanol and acetic acid were acquired from Penta (Prague, Czech Republic) and methanol-HPLC from LabScan (Sowińskiego, Polsko). Standards of phenolic compounds for HPLC analyses were purchased from Sigma Aldrich (St. Louis, MO, USA), all of HPLC-grade. Standards of ascorbic acid and *d*-α-tocopherol succinate were obtained from AccuStandard (New Haven, CT, USA). Other used chemicals were of analytical grade from Sigma Aldrich (St. Louis, MO, USA).

### 2.4. Extraction Methods

The extractions were done following the protocols as described by Orsavová et al., 2019 [18] and Sytařová et al., 2020 [19]. Briefly, lyophilized fruit samples (0.5 g) were extracted using 10 mL of the extraction mixture of either water and methanol (70/30, *v*/*v*) to determine total contents of phenolics (TP), flavonoids (TF) and antioxidant activity (DPPH) or redistilled water/methanol/acetic acid (69/30/1, *v*/*v*/*v*) for HPLC analysis in screw-cap test tubes in a shaking water bath (Memmert GmbH + Co.KG, Schwabach, Germany) at 50 °C for 60 min. Finally, the extracts were centrifuged at 2430× *g* for 15 min (Velocity 13µ, Dynamica Scientific Ltd., Milton Keynes, UK) at room temperature. Vitamin C was extracted from lyophilized fruit samples (0.5 g) by 2.5 mL of a mobile phase—methanol/H_3_PO_4_/redistilled water (99/0.5/0.5, *v*/*v*/*v*) in screw-cap test tubes in a shaker LT 2 (Kavalier, Sázava, Czech Republic) for 10 min in the dark. The extracts were poured into 10 mL-volumetric flasks and filled with a mobile phase. Vitamin E was extracted from lyophilized fruit samples (1.0 g) by 2.5 mL of methanol in screw-cap test tubes in an ultrasonic bath PS 04,000 A (Notus-Powersonic, Vráble, SR) at 40 °C for 60 min. The extracts were poured into 10 mL-volumetric flasks and filled with methanol. Redistilled water was prepared using PURELAB Classic (ELGA, Lane End Business Park, High Wycombe, UK).

The extraction for the analysis of total monomeric anthocyanin content (TMAC) was performed according to the protocol as reported by Orsavová et al., 2019 [18]. Briefly, the extraction was conducted from lyophilized fruit samples (1.5 g) by 5 mL of the mixture of methanol/water/acetic acid (70/29/1, *v*/*v*/*v*) in screw-cap test tubes in a shaking water bath (Memmert GmbH + Co.KG, Schwabach, Germany) at 50 °C for 60 min and subsequently in an ultrasonic bath at 40 °C for 60 min. The extracts were centrifuged at 3280× *g* for 15 min (Velocity 13µ, Dynamica Scientific Ltd., Milton Keynes, UK) at room temperature.

Prior to the analyses, all extracts and supernatants were filtrated using nylon micro-filters (SYRINGE, Cronus Syringe Filter, Nylon 13 mm × 0.45 μm, Labicom, Olomouc, Czech Republic).

### 2.5. Total Phenolic (TP) and Flavonoid (TF) Content Assay

Total phenolic content (TP) was determined using the Folin-Ciocalteu method and total flavonoid content (TF) using NaNO_2_, AlCl_3_·6H_2_O and NaOH. Both methods were conducted following the protocols described by Orsavová et al., 2019 [18] using UV/VIS spectrometer Lambda 25 (PerkinElmer, Waltham, MA, USA). The results were expressed as grams of gallic acid equivalent kg^−1^ of dry weight (g GAE kg^−1^ dw) for TP and as grams of rutin equivalent.kg^−1^ (g RE kg^−1^ dw) for TF.

### 2.6. Total Monomeric Anthocyanin Content (TMAC) Assays

Total monomeric anthocyanin content (TMAC) was determined by pH differential absorbance method (AOAC official method 2005.02) conducted according to Lee et al., 2005 [20]) using UV/VIS spectrometer Lambda 25 (PerkinElmer, Waltham, MA, USA). The results were expressed as grams of cyanidin-3-*O*-glucoside equivalent (g COG. kg^−1^ dw; molecular weight = 449.2 g mol^−1^, molar extinction coefficient = 26,900 L cm^−1^ mol^−1^).

### 2.7. Vitamins C and E Assays

Determination of vitamins C and E contents was provided using methods described by Orsavová et al., 2019 [18] and Sytařová et al., 2020 [19] using HPLC analysis system UltiMate^®^ 3000 (Dionex, Sunnyvale, CA, USA) with a diode-array detector (DAD). Briefly, vitamin C assay employed Acclaim 120 C8 (Dionex, MA, USA), the reverse-phase column with dimensions of 150 × 2.1 mm and particle size of 5 μm. The analysis conditions were as follows: the mixture of methanol/H_3_PO_4_/r-H_2_O in the ratio of 99:0.5:0.5 (*v*/*v*/*v*) was used as a mobile phase in an isocratic mode, the flow rate was 0.8 mL min^−1^, injection volume 20 μL, column temperature was maintained at 25 °C during the run and the time of analysis was 10 min. Chromatograms were registered at 275 nm. Determination of vitamin E employed Kinetex C-18 (Phenomenex, Torrance, CA, USA), the column with dimensions of 150 × 4.6 mm and particle size of 2.6 μm. The analysis conditions were as follows: the mixture of methanol (HPLC)/r-H_2_O in the ratio of 95:5 (*v*/*v*) was used as a mobile phase in an isocratic mode, the flow rate was 1 mL min^−1^, injection volume 20 μL, column temperature was maintained at 30 °C during the run and the time of analysis was 20 min. Chromatograms were registered at 230 nm. The quantification of vitamins C and E contents was calculated from calibration curves with ascorbic acid and *d*-alpha-tocopherol succinate as standards and the results were expressed in g kg^−1^ dw and in mg kg^−1^ dw, respectively.

### 2.8. Phenolic Compounds by HPLC Assays

Contents of individual phenolic compounds were established using HPLC device UltiMate^®^ 3000 (Dionex, Sunnyvale, CA, USA) with a diode-array detector (DAD) and Kinetex column C-18 (Phenomenex, Torrance, CA, USA) following the protocol by Orsavová et al., 2019 [18]. Briefly, the solvents were (A) the mixture of water/acetic acid in the ratio of 99:1 (*v*/*v*) and (B) water/acetonitrile/acetic acid in the ratio of 67:32:1 (*v*/*v*/*v*) with the gradient mode (0–10 min: 90% A + 10% B; 10–16 min: 80% A + 20% B; 16–20 min: 60% A + 40% B; 20–25 min: 50% A + 50% B; 25–27 min: 60% A + 40% B; 27–35 min: 90% A + 10% B). The analysis conditions were set as follows: the flow rate was 1 mL min^−1^, injection volume 10 μL, column temperature was maintained at 23 °C during the run and the time of analysis was 35 min. Chromatograms were registered at 275 nm. The identification of individual phenolic compounds was provided using the retention times and method of standard addition. Data signals were processed by LC ChromeleonTM 7.2 Chromatography Data System (Dionex, Sunnyvale, CA, USA).

Further parameters of calibrations for the used phenolics standards and vitamins are shown in Table A2.

### 2.9. Antioxidant Activity by DPPH and PCL Assays

Determination of antioxidant activities (AOA) was performed using DPPH (2,2-diphenyl-1-picrylhydrazyl; Sigma Aldrich, MO, USA) and PCL assays (antioxidant activity of water-soluble compounds—ACW and lipid-soluble compounds—ACL) according to the methods reported by Orsavová et al., 2019 [18]. Regarding the DPPH assay, the absorbance was measured at 515 nm using Lambda 25 (PerkinElmer, Waltham, MA, USA). Trolox (Sigma Aldrich, MO, USA) was applied as a standard and the results were expressed as grams of Trolox equivalent kg^−1^ (g Trolox kg^−1^ dw). PCL assays were conducted using PHOTOCHEM (Analytik Jena AG, Jena, Germany) following ACW and ACL set protocols applying ACW and ACL kits (Analytik Jena AG, Jena, Germany). The quantification of ACW and ACL was executed using the calibration curves with ascorbic acid (ACW) and Trolox (ACL) as standards and the results were expressed as grams of ascorbic acid equivalent kg^−1^ (g AA kg^−1^ dw) or Trolox equivalent kg^−1^ (g Trolox kg^−1^ dw), respectively.

### 2.10. Statistical Analysis

The results were expressed as means and standard deviations (SD). All analyses were executed three times to verify the significant difference between the measured values and evaluated using SPSS 12.0 (SPSS Inc., Chicago, IL, USA). A Shapiro–Wilk test was performed to confirm a normal data distribution. If data were normally distributed, one-way analysis of variance (Anova, Tukey’s test) with the significance level of *p* < 0.05 was applied; otherwise, a non-parametric Kruskal–Wallis test with the same significance level was performed. Pearson correlation coefficients (*r*) were calculated (Microsoft Office Excel 2013, Redmond, WA, USA) and the strength of correlations was evaluated using Evans’ classification [21].

## 3. Results and Discussion

### 3.1. Total Phenolic (TP), Flavonoid (TF), and Anthocyanin (TMAC) Contents

A significant impact of the cultivar, growing season and environmental conditions on the content of various bioactive compounds has been examined in fruit berries [22,23]. Similarly, evident differences in the total phenolic (TP), flavonoid (FL) and monomeric anthocyanin (TMAC) contents have been established reflecting the influence of the locality with different climatic conditions (Figure 1). The contents of TP, TF, and TMAC of eight cultivars of honeysuckle berries from two different areas are provided in Table 1.

The average contents of TP—40.76 g GAE kg^−1^ and TF—44.07 g RE kg^−1^ were higher in berries from Žabčice in comparison with TP—32.21 g GAE kg^−1^ and TF—42.51 g RE kg^−1^ in berries from Lednice. However, the average TMAC content of 3.39 g COG kg^−1^ in berries from Lednice (the area with more sunshine) was higher than 3.14 g COG kg^−1^ in berries from Žabčice (the area with more rain). Statistically, significant differences between the individual cultivars grown in the same locality have been monitored as well. In berries from Lednice, the lowest amounts of TP, TF and TMAC were found in Altaj—17.52 g GAE kg^−1^, 17.67 g RE kg^−1^ and 1.71 g COG kg^−1^, respectively; whereas the highest amounts of TP and TMAC were established in Maistar—47.75 g GAE kg^−1^ and 5.68 g COG kg^−1^, respectively. The highest amount of TF was determined in Fialka from both areas—61.43 g RE kg^−1^ (Lednice) and 60.44 g RE kg^−1^ (Žabčice). Concerning berries from Žabčice, the highest amounts of TP and TMAC were analyzed in the cultivar Amphora—54.08 g GAE kg^−1^ and 4.63 g COG kg^−1^, respectively. The contrasting lowest amounts of TP—28.41 g GAE kg^−1^ and TF—28.41 g RE kg^−1^ were detected in Leningradskij velikan; the cultivar Remont showed the lowest TMAC of 1.73 g COG kg^−1^. Finally, the group of medium early-ripening cultivars (Fialka, Leningradskij velikan, Kamchadalka and Remont) from Lednice appear to have lower TMAC contents in comparison with early and medium late-ripening cultivars, except for Altaj. Such an observation has not been monitored in Žabčice.

A great variability of TP, TF and TMAC contents in honeysuckle berries has been documented in connection with the influence of the geographical location, its specific climatic conditions, the year of harvest and type of cultivar. A higher TP content of 55.6 g GAE kg^−1^ in berries grown in Russia was published [24]. The obtained TP content in berries from Žabčice was in accordance with the published 8.32 g GAE kg^−1^ fresh weight (fw) [25] and 7.56 g GAE kg^−1^ fw [26], both originated in Canada. A very low TP content of 3.11 g GAE kg^−1^ fw in berries from Slovakia was reported [27]; and the lowest TP amount of 0.086 g GAE kg^−1^ fw was registered in berries from Northern China [22]. Considering the total flavonoid content (TF), Fialka from both areas showed similar amounts to 11.5 g QE kg^−1^ fw determined in berries from Canada [25]. Nevertheless, the lower TF content was recorded in the other analyzed cultivars from both studied areas. A significantly smaller TF content of 0.07 g RE kg^−1^ fw was reported in berries from Northern China [22].

Apart from the protection against biotic and abiotic stress, anthocyanins play a key role in the regulation of plant growth and development. They also function as plant pigments. However, their content is influenced by genetic factors, climatic conditions and varies during the ripening process [23,28,29,30]. That is why reported data and data obtained in this study describing TMAC contents in fruits are very inconsistent. Higher TMAC contents were found in berries from Lednice, a place with a low precipitation level, which is in accordance with recorded TMAC contents in Canadian berries also harvested in the year with a low amount of precipitation [31]. Nevertheless, their TMAC values were manifold times higher; for example, 87.5 g COG kg^−1^ in 2014 (more sunshine) and 29.0 g COG kg^−1^ in 2016 (more precipitation) in Morena berries if compared with the established values of 4.21 and 2.58 g COG kg^−1^ in Morena from Lednice and Žabčice, respectively. Furthermore, a smaller content of TMAC in berries harvested in Poland in 2005–2008 was monitored in an opposite trend than in this study—the highest TMAC content of 2.04 g COG kg^−1^ and 1.81 g COG kg^−1^ was reported in berries harvested in 2005 and 2006, the years with the high amount of precipitation, in contrast to 1.16 g COG kg^−1^ and 1.21 g COG kg^−1^ in berries harvested in 2007 and 2008, the years with a low precipitation level [32]. These divergences are remarkable since anthocyanins are synthesized via the phenylpropanoid pathway requiring a light stimulation of many enzymes and transcription factors which leads to higher anthocyanin contents [23,29]. Higher TMAC contents were published in berries of different cultivars grown in Russia—94.3 g COG kg^−1^ [24] as well as in berries from Canada—5.86 g COG kg^−1^ fw [26] and 1.94 g COG kg^−1^ fw [25]. Finally, considering the highest TMAC content, Amphora and Maistar were evaluated as the best cultivars from both areas.

### 3.2. Vitamin C and E Content

Vitamin C is an important signaling molecule with different pathways of its synthesis depending on a particular cell specialization. That explains its considerable content in plant bodies reflecting the type of plant tissue, genetic diversity and environmental conditions including the light intensity and harvest time. Great vitamin C contents have been reported in connection with a higher light intensity [11,17,18,19]. In accordance with these facts, as Table 1 shows, the higher average content of vitamin C of 24.02 g kg^−1^ was recorded in berries from Lednice, the area with a higher amount of sunshine, when compared with the amount of 20.83 g kg^−1^ in berries from Žabčice. Statistically significant differences between the cultivars in both areas were established. The values ranged from 18.41 (Altaj) to 28.55 g kg^−1^ (Maistar) in berries from Lednice and between 13.51 (Remont) and 27.15 g kg^−1^ (Amphora) in berries from Žabčice. Interestingly, the specific features of the locality seem to be an important factor influencing the vitamin C content in the medium early cultivars of Leningradskij velikan, Kamchadalka and Remont and medium late Maistair from Žabčice, the area with higher precipitation during the growing period. Such amounts of rain led to lower amounts of vitamin C when compared to the values of vitamin C in the same cultivars from Lednice. That is in accordance with published data [33] stating that late-crop cultivars performed a significant decrease of L-ascorbic content by 27% and 33% in Polish cultivars Brazova and Wojtek, respectively. However, early ripening cultivars showed inconsistent amounts of vitamin C in connection with the locality. A considerably high amount of 25.76 g kg^−1^ was established in Morena from Lednice contrasting to 16.28 g kg^−1^ monitored in the same cultivar from Žabčice. Nevertheless, the values in Altaj were the lowest in all plants from Lednice while the amount of 23.10 g kg^−1^ was detected in the same cultivar from Žabčice. Finally, Amphora with the highest amount of vitamin C—27.15 g kg^−1^ when considering the cultivars from Žabčice showed almost the identical amount of 27.19 g kg^−1^ in its plants from Lednice. Substantial differences in vitamin C contents have been reported: a low vitamin C content in the range of 1.78–4.21 g kg^−1^ in berries from Canada [31] and 0.34–0.41 g kg^−1^ in berries from Poland [32]; in both cases depending on the cultivar with the unconvincing effect of the climatic conditions. Similarly, low content of vitamin C was detected in berries grown in Portugal in the amount of 0.25 g kg^−1^ fw [4], as well as in berries from Poland ranging between 0.03–0.32 g kg^−1^ dw [34] and 0.09–0.30 g kg^−1^ fw [1]. Finally, great differences in vitamin C contents varying between 0.29 and 1.87 g kg^−1^ fw were published [7].

Considering vitamin E, its content may reflect the specific characteristics of the locality. As can be seen in Table 1, the higher average content of 2.35 mg ^−1^ was established in berries from Lednice when compared with the content of 1.73 mg kg^−1^ in berries from Žabčice. Concerning the cultivars from Lednice, the highest amount of 3.70 mg kg^−1^ was detected in Altaj. What is more, the content of vitamin E in all cultivars from Lednice was higher than it was established in the same cultivars from Žabčice; with the only exception of Leningradskij velikan reaching the highest amount of 3.66 mg kg^−1^. The lowest contents of vitamin E were recorded in the late medium ripening cultivar Maistar from both areas: 1.59 mg kg^−1^ (Lednice) and 0.90 mg kg^−1^ (Žabčice) which contrasts with a significantly high content of vitamin E of 9.3 mg kg^−1^ fw in berries from Portugal [4].

In summary, noticeable correlations between the contents of vitamins C and E and the type of locality and time of maturity were established in honeysuckle berries. More sunshine during the growing period contributed to the highest content of vitamin C in medium-late ripening Maistar from Lednice which was contrasting to the lowest content of vitamin E in this cultivar from both areas. Additionally, more precipitation during the maturation period in Žabčice initiated the opposite effect—the medium early cultivars performed the lowest average amount of vitamin C. However, early ripening Amphora possessed almost the same content of vitamin C without any apparent impact of the locality. The medium early cultivars from both areas showed almost the same average content of vitamin E.

### 3.3. Phenolic Compounds by HPLC

**Figure 2:**
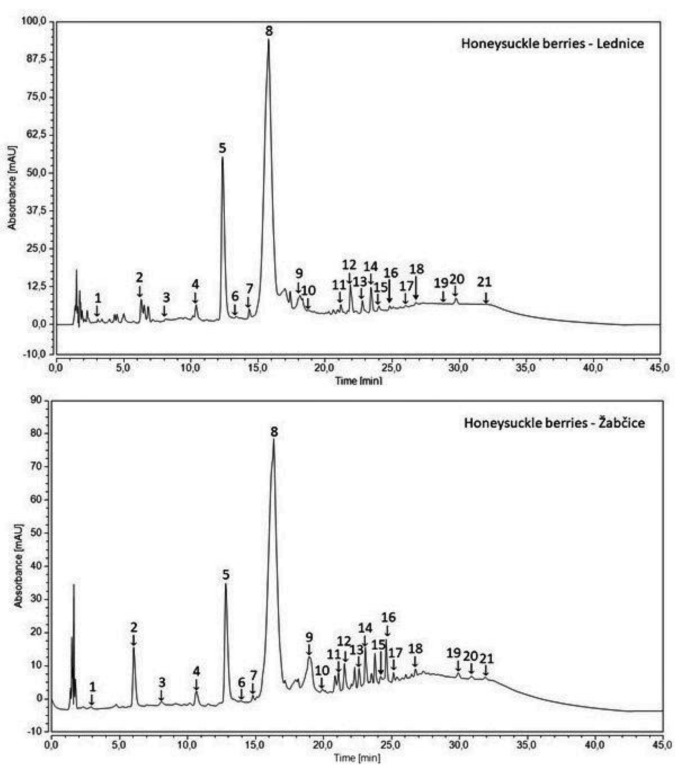
Characteristic chromatograms of honeysuckle berries from two localities of Lednice and Žabčice. Gallic acid (**1**), protocatechuic acid (**2**), neochlorogenic acid (**3**), 4-hydroxybenzoic acid (**4**), epigallocatechin (**5**), catechin (**6**), vanilic acid (**7**), chlorogenic acid (**8**), caffeic acid (**9**), syringic acid (**10**), epicatechin (**11**), *p*-cumaric acid (**12**), ferulic acid (**13**), sinapic acid (**14**), ellagic acid (**15**), rutin (**16**), *t*-cinnamic acid (**17**), protocatechuic acid ethylester (**18**), resveratrol (**19**), quercetin (**20**), hydroxycinnamic acid (**21**).

Characteristic chromatograms of individual phenolic compounds in honeysuckle berries from diverse localities are illustrated in Figure 2 and their profiles are depicted in Table 2 and Table 3. The composition of phenolic compounds has been very changeable. Evidently, the cultivars from Lednice were richer in total contents of quantified phenolic compounds (total-PP) with the highest total-PP of 6244.3 mg kg^−1^ detected in Amphora. The only exception was represented by Altaj showing almost a two-fold lower amount of 2781.3 mg kg^−1^ when compared with 4824.8 mg kg^−1^ in the same cultivar from Žabčice. However, such a variability of phenolic content is not influenced only by the specific locality, but genetic diversity may affect it as well. In accordance with this fact, Fialka showed a similar phenolic composition from both areas: 5939.0 mg kg^−1^ (Lednice) and 6036.8 mg kg^−1^ (Žabčice); the value of its phenolic content from Žabčice was also the highest among the cultivars from this area. The lowest total-PP of 3563.2 mg kg^−1^ in Žabčice was determined in Remont. Phenolic acids formed the major part of the total-PP, and particularly hydroxycinnamic acid derivatives, as important flavor precursors. The total-DCA (derivatives of cinnamic acid) content varied from 2419.2 mg kg^−1^ in Altaj to 5745.3 mg kg^−1^ in Amphora from Lednice and from 2968.9 mg kg^−1^ in Remont to 5345.0 mg kg^−1^ in Fialka from Žabčice.

In this study, CHL predominantly occurred in all cultivars regardless of the locality and varied between 2123.1 mg kg^−1^ in Altaj and 4770.8 mg kg^−1^ in Amphora from Lednice and between 2566.0 mg kg^−1^ in Amphora and 4654.9 mg kg^−1^ in Fialka from Žabčice. In accordance with the factors mentioned above, inconsistent contents of CHL in honeysuckle berries from different localities have been published: in Amphora 944.42 mg kg^−1^ and Leningradskij velikan 897.22 mg kg^−1^ originating in Lithuania [3], a wide range of 766.3–2940.1 mg kg^−1^ in berries from Poland [34], a range of 350–440 mg kg^−1^ fw in berries from Egypt [35], 207–327 mg kg^−1^ in berries from Canada [25], and similar differences in Amphora—364.3 mg kg^−1^, Fialka—584.7 mg kg^−1^, Kamtschadalka—227.6 mg kg^−1^, Leningradskij velikan—467.8 mg kg^−1^ and Morena—468.2 mg kg^−1^ fw from Poland [2]. Moreover, very low and variable CHL contents in Morena berries of 4.75 mg kg^−1^ (2014) and 2.85 mg kg^−1^ (2016) were published reflecting different harvest years [31]. Interestingly, decreasing amounts of CHL were reported in two cultivars of Evie (from 70.1 to 50.4 mg 100 g^−1^ fw) and Larissa (from 74.2 to 45.9 mg 100 g^−1^ fw) in connection with five different harvest times during two weeks [28]. In contrast, very high CHL contents ranging between 41,500–62,400 mg kg^−1^ in seven cultivars from China were documented [36]. Similarly, high contents of 17,750 mg kg^−1^ in leaves and 16,960 mg kg^−1^ in flowers of *Lonicera japonica* Thunb were reported [5].

Furthermore, *p*-cumaric acid (CU) and caffeic acid (CA) were the second and third dominantly presented DCAs in the analyzed samples. However, rather than the influence of the locality, statistical differences between the cultivars were established. In the cultivars from Lednice, the lowest amount of CU of 71.8 mg kg^−1^ was found in Leningradskij velikan and the highest of 762.0 mg kg^−1^ in Morena, while in the cultivars from Žabčice, CU contents ranged from 230.0 mg kg^−1^ in Remont to 770.1 mg kg^−1^ in Amphora. CA was presented in smaller amounts than CU, with the exception of Leningradskij velikan from Lednice. The lowest CA contents were recorded in Maistar—22.3 mg kg^−1^ (Lednice) and Morena—69.5 mg kg^−1^ (Žabčice). The highest amount, on the other hand, was detected in Amphora from both areas—226.4 mg kg^−1^ (Lednice) and 122.9 mg kg^−1^ (Žabčice) and almost the same amount of 122.8 mg kg^−1^ also in cultivar Fialka from Žabčice. Correspondingly, a higher CU content of 987.1 mg kg^−1^ in comparison to the CA amount of 598.2 mg kg^−1^ in berries from Poland was published [37]. Likewise, the CA content of 5.98 mg kg^−1^ fw was lower when compared to 9.87 mg kg^−1^ fw of CU in berries from Slovakia [27]. In contrast, higher CA contents of 67.76–143.17 mg kg^−1^ compared to CU amounts of 9.50–25.11 mg kg^−1^ were reported in berries from Lithuania [3]. Very low CU contents of 1–2 mg kg^−1^ fw were presented in berries from Canada [25]. Moreover, sinapic acid (SP) was determined in the range from 21.7 mg kg^−1^ (Altaj) to 109.9 mg kg^−1^ (Leningradskij velikan) in Lednice and from 14.4 mg kg^−1^ (Maistar) to 86.5 mg kg^−1^ (Kamchadalka) in Žabčice. However, in berries from Poland, SP was not detected at all [37]. Other phenolic acids from the DCA group were determined only in insignificant amounts.

Considering derivatives of benzoic acid, only small amounts were present. Nevertheless, in all cultivars from Lednice, the total content of derivatives of benzoic acid (total-DBA) was higher, except for Leningradskij velikan. The lowest total-DBA content was established in Remont from both areas: 107.2 mg kg^−1^ (Lednice) and 74.2 mg kg^−1^ (Žabčice). Contrastingly, Fialka from both localities possessed the highest total-DBA amount of 296.4 mg kg^−1^ (Lednice) and 241.7 mg kg^−1^ (Žabčice). Considering individual DBA, protocatechuic acid (PK) prevailed in almost all cultivars from Lednice varying from 36.7 mg kg^−1^ (Kamchadalka) to 196.0 mg kg^−1^ (Fialka). The only exception was Amphora and Kamchadalka with 4-hydroxybenzoic acid (HB)—86.1 mg kg^−1^ and ethyl ester protocatechuic acid (PKEE)—44.0 mg kg^−1^ recorded as predominant, respectively. Nonetheless, DBA contents established in the cultivars from Žabčice were more changeable; mainly, PK prevailed from 15.9 mg kg^−1^ in Remont to 163.8 mg kg^−1^ in Leningradskij velikan. In Amphora, HB with the amount of 36.4 mg kg^−1^ was established as the abundant DBA, likewise in the same cultivar from Lednice. Gallic acid (GA) was recorded in the prevalent content in Kamchadalka—49.6 mg kg^−1^ and Remont—28.4 mg kg^−1^, and PKEE with the amount of 26.0 mg kg^−1^ in Maistar. In accordance with these analyzed results, similar amounts of PK—144.4 mg kg^−1^ and GA—44.3 mg kg^−1^ were reported in berries from Poland, nonetheless, with a higher content of vanilic acid (VA) 21.1 mg kg^−1^ [37].

Flavonoid contents may be strongly affected by genetic heritage and epigenetic modification as mechanisms of plant responses to environmental stress [35]. Qi et al., 2020 reported dynamic changes and significant differences in the accumulation of flavonoids during the development stages of *Lonicera maackii* fruit [38]. Quercetin and rutin as the most common flavonoids show therapeutic effects; the former influences lipid accumulation and eliminates inflammation in non-alcoholic fatty liver, the other can attenuate neuroinflammation, improve memory deficits and delay pathological processes of Alzheimer’s disease [38]. Catechins also provide several health benefits by scavenging free radicals and directly affect the properties of the skin by activating synthesis of collagen [39]. In addition to the listed possible genetic factors, the specific locality seems to be one of the significant factors affecting total flavonoid contents (total-FL). Total-FL contents in the cultivars from Žabčice were determined in higher amounts than in the cultivars from Lednice and ranged in dependence on the particular cultivar from 105.1 mg kg^−1^ in Altaj to 519.7 mg kg^−1^ in Remont, except for two cultivars from Lednice—Altaj (153.8 mg kg^−1^) and Maistar with the highest total-FL of 988.1 mg kg^−1^. As far as flavonoids are concerned, flavanol epigallocatechin (EGC) was established as the predominant flavonoid substance in almost all cultivars from Žabčice and ranged from 30.9 mg kg^−1^ in Kamchadalka to 395.7 mg kg^−1^ in Remont, with the exception of Kamchadalka with flavonol rutin (RU) in the highest amount of 122.3 mg kg^−1^. Regarding cultivars from Lednice, representation of flavonoids was rather inconsistent. In five cultivars, EGC was predominant in the range from 31.7 mg kg^−1^ in Leningradskij velikan to 779.7 mg kg^−1^ in Maistar, whereas RU prevailed in four cultivars ranging from 29.9 mg kg^−1^ in Remont to 147.8 mg kg^−1^ in Fialka. Similarly, great differences in RU content were determined in berries of Morena varying from 3.67 mg kg^−1^ (2014) to 1.60 mg kg^−1^ (2016) reflecting variable harvest years [31]. Moreover, a high content of RU of 255.78–779.31 mg kg^−1^ was reported in berries from Lithuania [3]. Furthermore, significantly high contents of RU of 4400–15,100 mg kg^−1^ were documented in seven cultivars from China [36]; and also the amounts of 13,820 mg kg^−1^ in the leaves and 2500 mg kg^−1^ in the flowers of *Lonicera japonica* Thunb were reported [5]. Concerning other flavanols, epicatechin (EC) was established in the range from 5.0 mg kg^−1^ (Kamchadalka) to 53.0 mg kg^−1^ (Remont) in the cultivars from Lednice and from 7.5 mg kg^−1^ (Morena) to 99.4 mg kg^−1^ (Fialka) in the cultivars from Žabčice. Catechin (C) varied from 5.3 mg kg^−1^ (Altaj) to 93.0 mg kg^−1^ (Maistar) in the cultivars from Lednice and from 8.5 mg kg^−1^ (Leningradskij velikan) to 81.3 mg kg^−1^ (Remont) in the cultivars from Žabčice. Comparable EC amounts of 10.35–41.99 mg kg^−1^ were recorded in berries from Poland; however, C contents were higher in the range of 22.15–136.14 mg kg^−1^ [34]. Correspondingly, Kucharska et al., 2017 monitored the content of C in berries from Poland in the range of 21.4–312.2 mg kg^−1^ [2]. Moreover, higher EC and C amounts were reported in berries from Canada in the range of 7–71 mg kg^−1^ fw and of 17–54 mg kg^−1^ fw, respectively [25]. Furthermore, a different impact of various harvest times conducted in two subsequent weeks on the contents of C and EC in two cultivars of Evie and Larissa was reported [28]. While C content decreased from 4.79 to 1.96 mg 100 g^−1^ fw in Evie and from 7.84 to 4.99 mg 100 g^−1^ fw in Larissa, EC content increased from 4.97 to 5.57 mg 100 g^−1^ fw in Evie and from 1.83 to 2.93 mg 100 g^−1^ fw in Larissa. In contrast to the cultivars analyzed in this study, a high amount of quercetin (QUE) between 47.25 and 143.30 mg kg^−1^ was reported [3].

Finally, stilbene resveratrol (RES) was presented only in small amounts. The lowest content was recorded in Remont with the value of 0.5 mg kg^−1^ in berries from Lednice and 0.4 mg kg^−1^ from Žabčice. On the other hand, the highest contents were detected in Amphora from Lednice with the value of 5.5 mg kg^−1^ and in Morena and Leningradskij velikan from Žabčice with the same amount of 3.3 mg kg^−1^.

This study has proved the fact that the composition of phenolic compounds fluctuates. As has been presented, commonly higher contents of quantified total-PP were detected in the cultivars from Lednice. Such a variability of phenolic contents may stem from a number of factors including a significant effect of genetic diversity. Thus, Fialka from both areas showed similar total-PP contents regardless of the locality and seemed to be the most valuable cultivar considering the high contents of phenolic compounds. Phenolic acids generally form the main part of total-PP. In this study, particularly CHL predominantly occurred in all cultivars irrespective of the locality. Finally, considering the total average amount of phenolic compounds, medium early cultivars from both areas performed the highest abundance.

### 3.4. Antioxidant Activity (AOA)

Due to the occurrence of diverse phenolic compounds and vitamin C with different antioxidant effects in the plant matter, it seems beneficial to use different AOA assays to gain sufficient information about the antioxidant potential of various cultivars.

As can be seen in Table 4, the AOA values obtained by DPPH, ACW and ACL assays showed statistically significant differences in connection with the applied method, type of the cultivar and locality. The DPPH values seem to be influenced by the specific area the least since the average DPPH value of 44.48 g Trolox kg^−1^ in the cultivars from Lednice was only slightly higher than the amount of 43.33 g Trolox kg^−1^ in the cultivars from Žabčice. However, the average value of 29.45 g AA kg^−1^ obtained by ACW in the cultivars from Lednice was almost two-fold lower when compared to 51.84 g AA kg^−1^ in the cultivars from Žabčice. Concerning ACL assay, berries from Lednice showed a higher average value of 47.15 g Trolox kg^−1^ than 38.33 g Trolox kg^−1^ in berries from Žabčice.

Regarding DPPH, the highest values were obtained in Maistar from Lednice—59.31 g Trolox kg^−1^ and Morena from Žabčice—53.02 g Trolox kg^−1^. Morena and Amphora from both areas performed similarly high AOA values. The lowest DPPH values were observed in Altaj—27.35 g Trolox kg^−1^ from Lednice and in Remont—30.76 g Trolox kg^−1^ from Žabčice. Concerning the ACW method, the lowest values were monitored in the cultivars from Lednice reaching from 15.83 g AA kg^−1^ in Remont to 55.21 g AA kg^−1^ in Maistar which is in contrast to higher ACW values in the cultivars from Žabčice ranging between 34.13 g AA kg^−1^ in Maistar and 77.78 g AA kg^−1^ in Fialka. On the other hand, ACL values were the highest in the cultivars from Lednice when compared with the AOA values determined by the other methods and ranged from 29.51 g Trolox kg^−1^ in Altaj to 67.93 g Trolox kg^−1^ in Amphora. In the cultivars from Žabčice, ACL values were the lowest in comparison with the other methods and reached from 24.06 g Trolox kg^−1^ in Maistar to 52.40 g Trolox kg^−1^ in Amphora.

Evaluating the AOA of the cultivars from Lednice, the highest antioxidant potential was determined in Morena, Amphora and Maistar; Altaj showed the least significant values. Considering the AOA of the cultivars from Žabčice, Morena, Amphora and Fialka showed the most significant potential, while Maistar and Remont were the worst. In accordance with the DPPH values established in Morena—53.02 g Trolox kg^−1^ (Žabčice) and 53.56 g Trolox kg^−1^ (Lednice), the comparable value of 55.06 g Trolox kg^−1^ in Morena from Canada harvested in 2014 was published; however, with a great difference depending on the harvest year as only 26.28 g Trolox kg^−1^ was established in the same cultivar in 2016 [31]. Furthermore, lower DPPH values in Amphora—4.15 g Trolox kg^−1^ fw were reported [2] and 5.0 g Trolox kg^−1^ fw [1]. In spite of this fact, Amphora was evaluated as the cultivar with the highest antioxidant potential in both studies.

### 3.5. The Impact of the Monitored Factors on the Antioxidant Activity

Generally, AOA may be influenced by many factors, including the presence of diverse chemical compounds which could be further affected by the cultivar, part of the plant body, ripening and harvest time and locality. Furthermore, synergistic or antagonistic effects of these compounds play a crucial role in the resulting AOA. The evaluation of the impact of different factors on AOA was provided using Pearson correlation coefficients (r) at *p* < 0.05 and their values are shown in Table 5.

#### 3.5.1. The Impact of the Method of AOA Determination

Between the methods applied in AOA determination, different correlation coefficients (r) were observed with respect to the specific locality. Between DPPD and photochemiluminiscence methods of ACW and ACL in the cultivars from Lednice, very strong (*r =* 0.8288) and strong (*r* = 0.7348) correlations were monitored, respectively. Contrastingly, a moderate correlation with ACW (*r* = 0.5043) and significantly weak negative correlation with ACL (*r* = −0.0101) were established in Žabčice. Very strong correlations were reported between DPPH and ABTS radical scavenging activity (*r* = 0.96) and between DPPH and method of fluorescence recovery after photobleaching—FRAP (*r* = 0.99) [40]. Moreover, Gawroński et al., 2020 observed strong correlations between DPPH and ABTS (*r* = 0.7435) and (*r* = 0.6584) at the phenotype and genotype levels, respectively [1]. However, dissimilar correlations were established between various methods in berries from Canada, a very strong correlation between oxygen radical absorbance capacity ORAC and FRAP (*r* = 0.854) was determined contrasting to the weak correlations established between DPPH and ORAC (*r* = 0.021) and between DPPH and FRAP (*r* = −0.271) [41].

#### 3.5.2. The Impact of TP, TF and TMAC

As has been already stated, the distinct impact of TP, TF and TMAC on the resulting AOA in berries from various areas may be assumed from significantly strong correlations between TP and DPPH (*r* = 0.8257) and ACW (*r* = 0.8144) in the cultivars from Lednice. Otherwise, only moderate correlations were established. The findings of this study are consistent with very strong correlations between TP and ORAC (*r* = 0.95) and FRAP (*r* = 0.97) in berries from Oregon [42] and between TP and DPPH (*r* = 0.99), ABTS (*r* = 0.96) and FRAP (*r* = 0.96) [40].

Furthermore, they are in accordance with the correlations between TP and DPPH (*r* = 0.9417) and (*r* = 0.9520) at the phenotype and genotype levels, respectively [1]. Similarly, strong correlations were reported in berries from Poland between TP and ABTS (*r* = 0.620) and FRAP (*r* = 0.783) [34], as well as between TP and ORAC (*r* = 0.743) and FRAP (*r* = 0.867), yet in contrast to a negative weak correlation with DPPH (*r* = −0.368) [41].

Regarding TF, a very strong correlation was established with ACW (*r* = 0.8395) in the cultivars from Žabčice. Between TF and ACL in Žabčice, a very weak correlation was established, otherwise the correlations were moderate. Nevertheless, very strong correlations between TF and DPPH at the phenotype (*r* = 0.8302) and genotype (*r* = 0.8551) levels were determined [1], and also between TF and DPPH (*r* = 0.94), ABTS (*r* = 0.86) and FRAP (*r* = 0.91) [40]. Rupasinghe at al., 2012 reported very strong correlations between TF and ORAC (*r* = 0.907) and FRAP (*r* = 0.904) and a negative weak correlation with DPPH (*r* = −0.125) [41].

Concerning TMAC, a very strong correlation was observed between TMAC and DPPH (*r* = 0.9329) and between TMAC and ACW (*r* = 0.8926) in the cultivars from Lednice. Higher amounts of TMAC, vitamin C and CHL were recorded in the cultivars from Lednice performing higher DPPH values which is in accordance with data describing the cultivars from Canada [31]. A strong correlation was established between TMAC and DPPH (*r* = 0.6108) in the cultivars from Žabčice which corresponds with strong correlations between TMAC and DPPH (*r* = 0.78) and FRAP (*r* = 0.94) [2]. Furthermore, very strong correlations between TMAC and ORAC (*r* = 0.93) and FRAP (*r* = 0.95) were reported [42] and also strong correlations between TMAC and ABTS (*r* = 0.666) and FRAP (*r* = 0.732) [34].

#### 3.5.3. The Impact of Vitamins C and E

Vitamin C is an important component of honeysuckle berries proven to perform antioxidant activity. Regardless of the method of AOA determination, positive correlations were established between its content and AOA with higher correlation coefficients in the cultivars from Lednice, the area with more sunshine resulting in higher contents of vitamin C. Therefore, strong correlations were found with DPPH (*r* = 0.7884) and ACW (*r* = 0.7720) and moderate (*r* = 0.5755) with ACL in the cultivars from Lednice; while in the cultivars from Žabčice, there was established only a moderate correlation (*r* = 0.5196) with DPPH, weak (*r* = 0.2878) with ACW and very weak (*r* = 0.0651) with ACL. These results are in discrepancy with published the only negative weak correlation between vitamin C and ABTS (*r* = −0.246) and FRAP (*r* = −0.220) [34]. Further weak negative correlations between vitamin C and DPPH (*r* = −0.0751) and (*r* = −0.1911) were reported at the phenotype and genotype levels, respectively [1].

Considering vitamin E, variable and mostly negative values of correlation coefficients were calculated. The strong negative correlation was determined only between vitamin E and DPPH (*r* = −0.6051) in the cultivars from Lednice. However, in the cultivars from Žabčice, either negative or positive changeable weak correlations were established. The same or relatively low AOAs of 1.0 mM Trolox L^−1^ of vitamins C and E were reported [43]; nonetheless, the AOA potential of vitamins C and E by synergic activity of RU was recorded [14].

#### 3.5.4. The Impact of Individual Phenolic Compounds

Different pathways of metabolism and synergistic or antagonistic effects could influence the resulting antioxidant potential of individual phenolic compounds. Regarding flavonols, QUE performed strong positive correlations with DPPH (*r* = 0.6842) and ACW (*r* = 0.7423) in the cultivars from Lednice, while only a negative very strong correlation with ACL (*r* = −0.8062) in Žabčice. On the other hand, RU provided negative and weak correlations with DPPH, ACW and ACL, contrasting to the strong correlation (*r* = 0.747) with ABTS [3]. Moreover, Lee et al., 2019 reported a stronger correlation for RU and DPPH (*r* = 0.71) and FRAP (*r* = 0.74) than for RU with ABTS (*r* = 0.54) and for QUE with DPPH, FRAP and ABTS with *r* = 0.83, *r* = 0.87 and *r* = 0.75, respectively [40]. Strong correlations between flavonols and ORAC (*r* = 0.83) and FRAP (*r* = 0.82) were documented [42], and between flavonols and ABTS and FRAP weak (*r* = 0.307) and moderate (*r* = 0.404) correlations, respectively [34]. Among flavanols, strong correlations with AOAs were rare, positive strong between EGC and ACW (*r* = 0.6071) in the cultivars from Lednice, very strong between EC and ACW (*r* = 0.8303) from Žabčice and negative strong from Lednice (*r* = −0.6345). The last flavonol C correlated strongly only with DPPH (*r* = 0.6396) in the cultivars from Lednice; for the other relations, only weak positive or negative correlations were established. Stilben RES showed a very strong correlation only with ACL (*r* = 0.8193) and moderate with DPPH (*r* = 0.5170) in the cultivars from Lednice, in other cases, only weak correlations were established.

Concerning phenolic acids, strong correlations were confirmed rarely. Among DBA in the cultivars from Žabčice, strong correlations were found only between VA and ACL (*r* = 0.6684), negative between PKEE and ACL (*r* = −0.7844) and very strong with ACW (*r* = 0.8186). Regarding DBA in the cultivars from Lednice, strong correlations were determined between SI and DPPH (*r* = 0.6057) and ACW (*r* = 0.6802) and between HB and ACL (*r* = 0.6116). In the other samples from both areas, mostly weak or very weak correlations were established. Similarly, among DCA, strong correlations were seldom determined. FER showed a negative very strong correlation with ACL (*r* = −0.8191) in the cultivars from Žabčice. Further, CHL and NCHL strongly correlated with ACL, positively (*r* = 0.7850) in the cultivars from Lednice, negatively (*r* = −0.7960) from Žabčice. Finally, CU performed a strong correlation with DPPH (*r* = 0.6144) and a very strong with ACL (*r* = 0.8014) in the cultivars from Lednice, and a strong correlation with ACW (*r* = 0.7101) in the cultivars from Žabčice; otherwise, only moderate correlations were found. Inconsistent values of correlation coefficients between various methods of AOA and phenolic acids determination were reported. In accordance with the results of this study, CHL strongly correlated with ORAC (*r* = 0.828) and FRAP (*r* = 0.796) [42]. However, Lee et al., 2019 reported identical very strong correlations (*r* = 0.94) between CHL and DPPH and FRAP, and with ABTS (*r* = 0.88); CA performed very strong correlations with DPPH, FRAP and ABTS with *r* = 0.96, *r* = 0.97 and *r* = 0.89, respectively [40]. Raudonė et al., 2021) registered very strong correlations between cupric reducing activity (CuPRAC) and CA and FER with the same value of *r* = 0.833, with CHL (*r* = 0.881) and a strong correlation with CU (*r* = 0.714) [3]. However, variable correlations were published between phenolic acids and ABTS (*r* = 0.296) and FRAP (*r* = 0.575) [34].

The significant AOA of honeysuckle berries stems from the presence of high contents of polyphenols, especially flavonoids and cinnamic acids, whose phenolic groups can accept an electron to form relatively stable radicals, thus ceasing chain oxidation reactions in cellular components [44]. However, AOA may be affected by various interactions of the mixture of bioactive compounds. Interestingly, high AOA values for QUE (4.7 mM Trolox L^−1^) and RU (2.4 mM Trolox L^−1^) were published [43]. Therefore, such a presence of QUE may contribute to high AOA levels of honeysuckle berries. Strong synergic QUE effects have been established in the mixtures with other phenolic compounds, e.g., in the mixture of QUE, GA and CA of 59.4%, QUE, RU and GA of 55.2% and QUE and CA of 37.9%; however, the mixture of RU, CHL and CA performed the antagonistic effect of −15.8% [14]. Moreover, a high content of phenolic acids and their possible interactions with other compounds could play an important role in the significant AOA of honeysuckle berries. CHL was recorded as the prevalent DCA. Although its published AOA is rather low, the mixture of CHL, GA and CA showed a high synergic effect of 25.7%. Additionally, GA was monitored as the second most frequent DBA. Its binary mixtures showed a very strong synergic effect of 137.8% with CA and 27.9% with CHL [14].

## 4. Conclusions

Berries of *Lonicera caerulea* L. may be considered as a promising source of the main phenolic compounds and vitamins. However, as this study has confirmed, their contents may vary significantly depending on a number of external and internal factors including different climatic conditions of the particular locality and cultivar. Berries from Lednice, the area with more sunshine, showed higher average contents of almost all determined substances than berries from Žabčice, the area with higher precipitation levels, apart from the average TP and TF contents that reached higher amounts in berries from Žabčice. Furthermore, the statistically significant effect of various maturity times on chemical composition among the groups of early, medium early and medium late cultivars were observed. Regarding TMAC and vitamin C content, early ripening Amphora from both areas may be assessed as the best cultivar; concerning the content of phenolic compounds, Fialka from both areas and Amphora from Lednice may be considered as the most valuable.

What is more, the results of this study contribute to facilitate the selection of the most appropriate cultivars from the producers’ and consumers’ point of view. Since the fluctuation of bioactive compounds contents may be activated by complex epigenetic regulatory mechanisms of plant responses to different ambient conditions and environmental stress, cultivation of a greater number of cultivars with different maturing times is required to guarantee good-quality berries with high nutritional values.

## Figures and Tables

**Table 1:** Total contents of phenolics (TP) and flavonoids (TF), monomeric anthocyanins (TMAC), vitamins C and E contents in honeysuckle berries from two various locations.

Honeysuckle Cultivars	Phenolics (TP) [g GAE kg^−1^]			Flavonoids (TF) [g RE kg^−1^]			Monomeric Anthocyanins (TMAC) [g COG kg^−1^]			Vitamin C [g kg^−1^]			Vitamin E [mg kg^−1^]		
	Mean		SD	Mean		SD	Mean		SD	Mean		SD	Mean		SD
Lednice															
Morena	37.23	±	0.24 ^a,e,o^	55.69	±	0.60 ^a^	4.21	±	0.07 ^a^	25.76	±	0.05 ^a^	2.15	±	0.03 ^a^
Altaj	17.52	±	0.28 ^b^	17.67	±	0.15 ^b^	1.71	±	0.02 ^b^	18.41	±	0.60 ^b^	3.70	±	0.00 ^b^
Amphora	33.57	±	0.53 ^c^	33.54	±	0.59 ^c^	4.14	±	0.07 ^a^	27.19	±	0.01 ^c^	2.77	±	0.00 ^c^
Fialka	30.67	±	0.16 ^d^	61.43	±	0.24 ^d,l^	2.94	±	0.07 ^c^	21.26	±	0.10 ^d^	1.67	±	0.01 ^d^
Leningradskij velikan	33.53	±	0.62 ^c^	40.59	±	0.30 ^e,g,p^	2.52	±	0.06 ^d,h^	26.69	±	0.16 ^e^	2.46	±	0.06 ^e^
Kamchadalka	36.50	±	0.69 ^a,e,o^	42.73	±	0.41 ^f,i,p^	3.25	±	0.11 ^e^	24.48	±	0.24 ^f^	2.59	±	0.10 ^e^
Remont	28.87	±	0.99 ^f,l^	40.75	±	0.36 ^e,g,o,p^	2.68	±	0.10 ^d^	19.81	±	0.06 ^g^	1.88	±	0.02 ^f^
Maistar	47.75	±	0.04 ^g,l^	47.65	±	0.60 ^h^	5.68	±	0.31 ^f^	28.55	±	0.06 ^h^	1.59	±	0.01 ^g^
Average	32.21	±	0.49	42.51	±	0.41	3.39	±	0.10	24.02	±	0.16	2.35	±	0.03
Žabčice															
Morena	43.83	±	0.22 ^h,n^	43.52	±	0.51 ^f,i,n^	2.58	±	0.03 ^d,h^	16.28	±	0.13 ^i^	1.48	±	0.01 ^h^
Altaj	38.23	±	0.71 ^i,k^	38.66	±	0.48 ^j^	2.87	±	0.05 ^c^	23.10	±	0.22 ^j^	1.94	±	0.03 ^i^
Amphora	54.08	±	0.18 ^j^	54.11	±	0.13 ^k^	4.63	±	0.05 ^g^	27.15	±	0.23 ^c^	1.57	±	0.01 ^g^
Fialka	39.18	±	0.63 ^k^	60.44	±	0.88 ^d,l^	3.39	±	0.07 ^e^	23.43	±	0.27 ^j^	1.24	±	0.02 ^j^
Leningradskij velikan	28.41	±	0.46 ^f,l^	28.41	±	0.47 ^m^	2.98	±	0.07 ^c^	19.05	±	0.02 ^k^	3.66	±	0.03 ^b^
Kamchadalka	44.28	±	0.46 ^h,m^	44.35	±	0.36 ^i,n^	2.45	±	0.10 ^h^	18.85	±	0.03 ^b^	1.95	±	0.02 ^i^
Remont	41.51	±	0.70 ^n^	41.38	±	0.48 ^e,g,o,p^	1.73	±	0.03 ^b^	13.51	±	0.13 ^l^	1.07	±	0.04 ^k^
Maistar	36.57	±	0.59 ^a,e,o^	41.68	±	1.32 ^f,o,p^	4.49	±	0.39 ^a,g^	25.28	±	0.06 ^m^	0.90	±	0.01 ^l^
Average	40.76	±	0.49	44.07	±	0.58	3.14	±	0.10	20.83	±	0.14	1.73	±	0.02

The results are expressed in dry weight as means ± SD, *n* = 5. The results in the same column followed by the same letters do not significantly differ by Tukey’s test (*p* < 0.05).

**Table 2:** Content of phenolic compounds in honeysuckle berries from Lednice by HPLC.

Cultivars of Honeysuckle Berries from Lednice																									
Phenolics [mg kg^−1^]		Morena			Altaj			Amphora			Fialka			Leningradskij Velikan			Kamchadalka			Remont			Maistar		
		Mean		SD	Mean		SD	Mean		SD	Mean		SD	Mean		SD	Mean		SD	Mean		SD	Mean		SD
QUE		1.3	±	0.0 ^a^	0.6	±	0.0 ^b^	1.9	±	0.1 ^c^	0.9	±	0.1 ^d^	0.5	±	0.1 ^b^	1.2	±	0.0 ^e^	1.6	±	0.0 ^f^	5.4	±	0.1 ^g^
RU		43.2	±	0.3 ^a^	91.0	±	0.2 ^b^	38.1	±	0.7 ^c^	147.8	±	2.4 ^d^	42.9	±	0.1 ^a^	122.4	±	2.2 ^e^	29.9	±	0.7 ^f^	104.0	±	0.5 ^g^
EGC		45.4	±	3.9 ^a^	22.0	±	0.2 ^b^	151.4	±	2.0 ^c^	178.1	±	3.2 ^d^	31.7	±	0.5 ^e^	81.9	±	1.6 ^f^	241.7	±	5.0 ^g^	779.7	±	27.0 ^h^
EC		6.5	±	0.4 ^a^	34.1	±	0.3 ^b^	5.9	±	0.1 ^c^	10.5	±	0.0 ^d^	12.8	±	0.3 ^e^	5.0	±	0.1 ^f^	53.0	±	0.5 ^g^	5.9	±	0.3 ^a,c^
C		26.9	±	1.4 ^a^	5.3	±	0.1 ^b^	81.6	±	0.1 ^c^	56.9	±	0.3 ^d^	34.7	±	0.3 ^e^	27.4	±	0.4 ^a^	83.5	±	0.4 ^f^	93.0	±	2.2 ^g^
Total-FL		123.3	±	6.0 ^a^	153.8	±	4.0 ^b^	278.8	±	2.9 ^c^	394.2	±	6.0 ^d^	122.6	±	1.2 ^a^	237.8	±	4.4 ^e^	409.7	±	6.6 ^f^	988.1	±	30.0 ^g^
Stilbene RES		2.0	±	0.1 ^a^	1.2	±	0.1 ^b^	5.5	±	0.0 ^c^	3.3	±	0.1 ^d^	1.1	±	0.0 ^b^	2.0	±	0.0 ^e^	0.5	±	0.0 ^f^	1.9	±	0.0 ^g^
GA		30.8	±	0.9 ^a^	10.8	±	0.0 ^b^	12.9	±	0.2 ^c^	29.1	±	0.3 ^d^	33.7	±	0.3 ^e^	31.8	±	0.9 ^a^	38.2	±	0.3 ^f^	20.0	±	0.6 ^g^
VA		9.2	±	0.0 ^a^	1.8	±	0.1 ^b^	2.9	±	0.0 ^c^	3.4	±	0.1 ^d^	7.9	±	0.1 ^e^	4.2	±	0.1 ^f^	3.0	±	0.1 ^c^	4.3	±	0.1 ^f^
SI		1.9	±	0.5 ^a,f^	2.1	±	0.0 ^a,b^	2.9	±	0.0 ^c^	3.6	±	0.3 ^d^	2.7	±	0.4 ^c,e^	2.1	±	0.3 ^a,b,e^	1.5	±	0.0 ^f^	8.5	±	1.8 ^g^
PK		56.2	±	0.4 ^a^	164.5	±	1.1 ^b^	68.3	±	0.2 ^c^	196.0	±	0.5 ^d^	91.2	±	0.8 ^e^	36.7	±	0.3 ^f^	41.0	±	0.5 ^g^	52.0	±	0.6 ^h^
PKEE		16.3	±	1.0 ^a^	9.1	±	0.1 ^b^	41.6	±	0.4 ^c^	47.1	±	0.5 ^d^	20.0	±	0.6 ^e^	44.0	±	0.8 ^f^	8.8	±	0.3 ^b^	27.7	±	3.0 ^g^
HB		13.8	±	0.0 ^a^	18.7	±	0.1 ^b^	86.1	±	1.1 ^c^	17.2	±	0.3 ^d^	29.4	±	0.1 ^e^	13.3	±	0.0 ^f^	14.4	±	0.2 ^g^	11.0	±	0.0 ^h^
EL		0.0	±	0.0 ^a^	0.0	±	0.0 ^a^	0.1	±	0.0 ^b^	0.0	±	0.0 ^a^	0.1	±	0.0 ^b^	1.7	±	0.0 ^c^	0.2	±	0.0 ^d^	0.0	±	0.0 ^a^
Total-DBA		128.2	±	2.9 ^a^	207.1	±	1.4 ^b^	214.7	±	2.0 ^c^	296.4	±	2.0 ^d^	185.0	±	2.3 ^e^	133.9	±	2.4 ^f^	107.2	±	1.4 ^g^	123.5	±	6.1 ^a^
CN		12.0	±	0.0 ^a^	13.5	±	0.0 ^b^	11.4	±	0.1 ^c^	9.3	±	0.0 ^d^	11.2	±	0.2 ^c^	5.8	±	0.1 ^e^	12.6	±	0.7 ^a^	10.4	±	0.3 ^f^
HCN		5.8	±	0.2 ^a^	12.2	±	0.2 ^b^	20.7	±	0.6 ^c^	19.7	±	0.3 ^d^	5.6	±	0.0 ^a^	18.0	±	0.6 ^e^	17.2	±	0.2 ^e^	13.8	±	0.1 ^f^
CA		66.1	±	0.0 ^a^	94.7	±	0.9 ^b^	226.4	±	3.5 ^c^	112.6	±	3.7 ^d^	101.8	±	1.9 ^e^	116.0	±	3.5 ^d^	163.8	±	0.9 ^f^	22.3	±	0.4 ^g^
FER		37.3	±	0.1 ^a^	24.0	±	0.1 ^b^	11.7	±	0.3 ^c^	24.4	±	0.1 ^d^	37.4	±	0.1 ^a^	70.7	±	0.1 ^e^	16.9	±	0.4 ^f^	1.2	±	0.0 ^h^
CHL		4128.5	±	3.1 ^a^	2123.1	±	7.8 ^b^	4770.8	±	13.0 ^c^	4537.3	±	15.7 ^d^	4278.9	±	1.2 ^e^	3260.6	±	6.1 ^f^	3325.7	±	4.4 ^g^	2886.8	±	4.3 ^h^
NCHL		21.1	±	0.1 ^a^	13.6	±	0.1 ^b^	6.6	±	0.2 ^c^	13.9	±	0.0 ^d^	21.2	±	0.0 ^a^	6.4	±	0.1 ^c^	3.6	±	0.0 ^e^	3.1	±	0.1 ^h^
CU		762.0	±	1.9 ^a^	116.3	±	0.7 ^b^	656.4	±	0.5 ^c^	495.6	±	1.4 ^d^	71.8	±	1.1 ^e^	676.9	±	3.8 ^f^	318.2	±	1.7 ^g^	460.3	±	2.4 ^h^
SP		33.4	±	0.2 ^a^	21.7	±	0.1 ^b^	41.3	±	0.3 ^c^	32.3	±	0.3 ^d^	109.9	±	1.5 ^e^	45.9	±	0.2 ^f^	24.6	±	0.1 ^g^	79.7	±	1.2 ^h^
Total-DCA		5066.3	±	5.7 ^a^	2419.2	±	9.9 ^b^	5745.3	±	18.5 ^c^	5245.1	±	21.5 ^d^	4637.9	±	6.1 ^e^	4560.4	±	14.4 ^f^	3882.8	±	4.9 ^g^	3617.7	±	11.1 ^h^
Total-PP	5319.7		±	14.7 ^a^	2781.3	±	15.4 ^b^	6244.3	±	23.4 ^c^	5939.0	±	29.6 ^d^	4946.5	±	9.7 ^e^	4934.1	±	21.3 ^e^	4400.2	±	12.8 ^f^	4731.2	±	47.2 ^g^

The results are expressed in dry weight as means ± SD, *n* = 5. The results in the same line followed by the same letter do not significantly differ by Tukey’s test (*p* < 0.05). Flavonoids (FL): QUE (quercetin), RU (rutin), EGC (epigallocatechin), EC (epicatechin), C (catechin); stilben RES (resveratrol). Kaempferol was not detected. Phenolic acids: GA (gallic), VA (vanilic), SI (syringic), PK (protocatechuic), PKEE (protocatechuic acid ethylester), HB (4-hydroxybenzoic), EL (ellagic), Total-DBA (total of benzoic acid derivatives), CN (*t*-cinnamic), HCN (hydroxycinnamic), CA (caffeic), FER (ferulic), CHL (chlorogenic), NCHL (neochlorogenic), CU (*p*-cumaric), SP (sinapic), Total-DCA (total of cinnamic acid derivatives), Total-PP (total phenolics).

**Table 3:** Content of phenolic compounds in honeysuckle berries from Žabčice by HPLC.

Cultivars of Honeysuckle Berries from Žabčice																								
Phenolics [mg kg^−1^]	Morena			Altaj			Amphora			Fialka			Leningradskij Velikan			Kamchadalka			Remont			Maistar		
	Mean		SD	Mean		SD	Mean		SD	Mean		SD	Mean		SD	Mean		SD	Mean		SD	Mean		SD
QUE	1.7	±	0.1 ^a^	0.1	±	0.0 ^b^	0.1	±	0.0 ^b^	1.3	±	0.2 ^c^	0.4	±	0.0 ^d^	2.2	±	0.1 ^e^	1.1	±	0.0 ^f^	8.1	±	0.0 ^g^
RU	32.8	±	0.4 ^a^	20.0	±	0.2 ^b^	45.9	±	0.0 ^c^	61.7	±	0.4 ^d^	105.8	±	0.9 ^e^	122.3	±	2.1 ^f^	21.5	±	0.2 ^g^	105.5	±	2.7 ^e^
EGC	140.7	±	3.0 ^a^	55.9	±	3.9 ^b^	155.0	±	0.6 ^c^	237.8	±	5.6 ^d^	129.6	±	5.5 ^e^	30.9	±	0.7 ^f^	395.7	±	12.1 ^g^	157.2	±	2.2 ^c^
EC	7.5	±	0.5 ^a^	17.7	±	0.1 ^b^	49.7	±	0.5 ^c^	99.4	±	0.0 ^d^	19.4	±	0.3 ^e^	66.4	±	1.0 ^f^	20.2	±	0.0 ^g^	24.1	±	0.3 ^h^
C	22.3	±	0.5 ^a^	11.5	±	0.8 ^b^	31.1	±	0.9 ^c^	48.7	±	1.8 ^d^	8.5	±	0.2 ^e^	56.0	±	0.4 ^f^	81.3	±	2.5 ^g^	31.1	±	0.9 ^c^
Total-FL	204.9	±	4.5 ^a^	105.1	±	5.0 ^b^	281.8	±	2.1 ^c^	448.9	±	8.0 ^d^	263.7	±	7.0 ^e^	277.8	±	4.4 ^f^	519.7	±	14.8 ^g^	326.1	±	6.1 ^h^
Stilbene RES	3.3	±	0.1 ^a^	1.1	±	0.1 ^b^	0.6	±	0.0 ^c^	1.2	±	0.0 ^d^	3.3	±	0.0 ^a^	1.7	±	0.0 ^e^	0.4	±	0.0 ^f^	1.0	±	0.1 ^b^
GA	15.8	±	0.2 ^a^	19.7	±	0.5 ^b^	20.9	±	0.6 ^c^	35.1	±	0.5 ^d^	16.4	±	0.0 ^e^	49.6	±	0.0 ^f^	28.4	±	0.8 ^g^	17.0	±	0.8 ^e^
VA	3.8	±	0.1 ^a^	5.9	±	0.2 ^b^	5.3	±	0.6 ^c^	2.2	±	0.2 ^d^	3.3	±	0.4 ^e^	4.1	±	0.3 ^a^	6.8	±	0.1 ^f^	2.3	±	0.0 ^g^
SI	6.3	±	0.1 ^a^	8.3	±	0.0 ^b^	3.2	±	0.4 ^c^	2.7	±	0.0 ^d^	1.2	±	0.1 ^e^	3.7	±	0.2 ^c^	2.6	±	0.3 ^d^	1.3	±	0.0 ^e^
PK	63.0	±	0.1 ^a^	24.7	±	0.1 ^b^	24.4	±	0.2 ^b,c^	136.4	±	0.8 ^d^	163.8	±	3.9 ^e^	21.6	±	1.3 ^f^	15.9	±	1.0 ^g^	24.1	±	0.2 ^c^
PKEE	20.0	±	0.0 ^a^	9.1	±	0.1 ^b^	2.1	±	0.0 ^c^	27.1	±	0.8 ^d^	13.8	±	0.0 ^e^	6.8	±	0.3 ^f^	1.8	±	0.0 ^g^	26.0	±	0.1 ^h^
HB	4.1	±	0.4 ^a^	14.9	±	0.5 ^b^	36.4	±	0.2 ^c^	38.0	±	3.1 ^d^	22.7	±	0.3 ^e^	29.0	±	0.4 ^f^	17.0	±	0.1 ^g^	10.8	±	0.3 ^h^
EL	0.7	±	0.1 ^a^	10.7	±	0.1 ^b^	1.6	±	0.1 ^c^	0.2	±	0.0 ^d^	0.0	±	0.0 ^e^	0.0	±	0.0 ^e^	1.7	±	0.1 ^c^	2.1	±	0.1 ^f^
Total-DBA	113.7	±	0.9 ^a^	93.3	±	1.7 ^b^	93.8	±	2.2 ^c^	241.7	±	5.3 ^d^	221.2	±	4.8 ^e^	114.8	±	2.7 ^a^	74.2	±	2.4 ^f^	83.7	±	1.6 ^b^
CN	8.8	±	0.0 ^a^	8.6	±	0.0 ^b^	12.0	±	0.0 ^c^	11.6	±	0.4 ^c^	9.3	±	0.0 ^d^	10.6	±	0.0 ^e^	8.6	±	0.0 ^b^	12.2	±	0.2 ^c^
HCN	4.3	±	0.1 ^a^	3.0	±	0.0 ^b^	6.4	±	0.0 ^c^	2.5	±	0.1 ^d^	14.0	±	0.0 ^e^	17.1	±	0.5 ^f^	3.1	±	0.0 ^g^	2.4	±	0.6 ^h^
CA	69.5	±	0.5 ^a^	110.8	±	2.0 ^b^	122.9	±	2.0 ^c^	122.8	±	0.6 ^c^	92.5	±	0.3 ^d^	93.3	±	1.1 ^d^	109.5	±	0.4 ^b^	119.3	±	0.4 ^e^
FER	25.0	±	0.2 ^a^	2.1	±	0.0 ^b^	1.1	±	0.0 ^c^	30.6	±	0.0 ^d^	15.9	±	0.3 ^e^	28.5	±	0.3 ^f^	0.7	±	0.0 ^g^	34.8	±	0.5 ^h^
CHL	2885.9	±	5.3 ^a^	4118.8	±	17.3 ^b^	2566.0	±	53.0 ^c^	4654.9	±	8.3 ^d^	2623.5	±	5.1 ^e^	3786.2	±	11.4 ^f^	2591.6	±	10.7 ^c^	3553.3	±	7.3 ^g^
NCHL	14.2	±	0.1 ^a^	1.2	±	0.0 ^b^	0.6	±	0.0 ^c^	13.7	±	0.1 ^d^	9.0	±	0.2 ^e^	16.2	±	0.2 ^f^	0.4	±	0.0 ^g^	19.1	±	0.6 ^h^
CU	318.5	±	5.3 ^a^	299.9	±	0.1 ^b^	770.1	±	1.4 ^c^	480.7	±	1.1 ^d^	447.2	±	12.5 ^e^	368.4	±	5.8 ^f^	230.0	±	3.5 ^g^	280.8	±	4.9 ^h^
SP	24.8	±	0.1 ^a^	80.9	±	0.3 ^b^	21.0	±	0.8 ^c^	28.3	±	0.5 ^d^	47.7	±	0.7 ^e^	86.5	±	0.5 ^f^	25.0	±	0.7 ^a^	14.4	±	0.3 ^g^
Total-DCA	3351.0	±	11.7 ^a^	4625.3	±	19.8 ^b^	3500.1	±	57.2 ^c^	5345.0	±	11.0 ^d^	3259.1	±	19.0 ^e^	4406.7	±	19.8 ^f^	2968.9	±	10.7 ^g^	4036.3	±	14.7 ^h^
Total-PP	3672.9	±	17.1 ^a^	4824.8	±	26.5 ^b^	3876.4	±	61.5 ^c^	6036.8	±	24.4 ^d^	3747.3	±	30.8 ^e^	4801.0	±	26.9 ^b^	3563.2	±	27.9 ^f^	4447.1	±	22.5 ^g^

The results are expressed in dry weight as means ± SD, *n* = 5. The results in the same line followed by the same letter do not significantly differ by Tukey’s test (*p* < 0.05). Flavonoids (FL): QUE (quercetin), RU (rutin), EGC (epigallocatechin), EC (epicatechin), C (catechin); stilben RES (resveratrol). Kaempferol was not detected. Phenolic acids: GA (gallic), VA (vanilic), SI (syringic), PK (protocatechuic), PKEE (protocatechuic acid ethylester), HB (4-hydroxybenzoic), EL (ellagic), Total-DBA (total of benzoic acid derivatives), CN (*t*-cinnamic), HCN (hydroxycinnamic), CA (caffeic), FER (ferulic), CHL (chlorogenic), NCHL (neochlorogenic), CU (*p*-cumaric), SP (sinapic), Total-DCA (total of cinnamic acid derivatives), Total-PP (total phenolics).

**Table 4:** Antioxidant activities of honeysuckle berries from two various locations.

Honeysuckle Cultivars	DPPH [g Trolox kg^−1^]			ACW [g AA kg^−1^]			ACL [g Trolox kg^−1^]		
	Mean		SD	Mean		SD	Mean		SD
Lednice									
Morena	53.56	±	0.01 ^a^	46.83	±	0.57 ^a^	60.29	±	1.93 ^a^
Altaj	27.35	±	0.09 ^b^	17.62	±	1.34 ^b^	29.51	±	1.38 ^b^
Amphora	54.64	±	0.04 ^c^	30.69	±	1.26 ^c^	67.93	±	0.74 ^c^
Fialka	44.54	±	0.03 ^d^	19.60	±	0.95 ^b^	50.77	±	1.15 ^d^
Leningradskii velikan	39.84	±	0.01 ^e^	25.58	±	2.09 ^d^	40.70	±	1.60 ^e,i,j^
Kamchadalka	37.92	±	0.05 ^f^	24.21	±	1.23 ^d^	46.20	±	0.20 ^f^
Remont	38.70	±	0.01 ^g^	15.83	±	0.45 ^b^	37.71	±	0.86 ^g,i^
Maistar	59.31	±	0.22 ^h^	55.21	±	1.22 ^e^	44.11	±	1.13 ^h^
Average	44.48	±	0.06	29.45	±	1.14	47.15	±	1.12
Žabčice									
Morena	53.02	±	0.31 ^i^	49.81	±	2.86 ^a^	37.90	±	1.56 ^e,g,i^
Altaj	38.70	±	0.01 ^g^	39.76	±	1.03 ^f^	44.38	±	1.14 ^h^
Amphora	51.48	±	0.12 ^h^	69.25	±	1.74 ^g^	52.40	±	1.62 ^d^
Fialka	49.78	±	0.09 ^i^	77.78	±	1.03 ^h^	31.25	±	0.70 ^b^
Leningradskii velikan	38.94	±	0.00 ^j^	43.95	±	1.65 ^i^	41.01	±	1.37 ^e,j^
Kamchadalka	38.37	±	0.01 ^k^	55.65	±	1.20 ^e^	36.91	±	1.49 ^g^
Remont	30.76	±	0.09 ^l^	44.43	±	1.96 ^a,i^	38.71	±	1.40 ^e,g,i^
Maistar	45.57	±	0.02 ^m^	34.13	±	0.46 ^j^	24.06	±	1.60 ^k^
Average	43.33	±	0.08	51.84	±	1.49	38.33	±	1.36

The results are presented in dry weight as means ± SD, *n* = 5. The results in the same column followed by the same letter do not significantly differ by Tukey’s test (*p* < 0.05). DPPH (2,2-diphenyl-1-picrylhydrazyl, ACW (water-soluble), ACL (lipid-soluble) antioxidant capacity.

**Table 5:** Antioxidant activities of honeysuckle berries from two various locations.

	Pearson Correlation Coefficients at *p* < 0.05					
	DPPH		ACW		ACL	
	Lednice	Žabčice	Lednice	Žabčice	Lednice	Žabčice
ACW	0.8288	0.5043	-	-	-	-
ACL	0.7348	−0.0101	0.4100	0.2405	-	-
TP	0.8257	0.3909	0.8144	0.5119	0.4326	0.4701
TF	0.5472	0.5702	0.3630	0.8395	0.4358	−0.0283
TMAC	0.9329	0.6108	0.8926	0.1831	0.5762	0.0609
Vitamin C	0.7884	0.5196	0.7720	0.2878	0.5755	0.0651
Vitamin E	−0.6051	−0.2307	−0.3898	−0.1336	−0.2395	0.3733
QUE	0.6842	0.1109	0.7423	−0.4243	0.0872	−0.8062
RU	−0.1211	−0.0471	−0.0596	−0.0713	−0.1818	−0.4396
Total flavonols	−0.0964	−0.0388	−0.0332	−0.0954	−0.1779	−0.4757
EGC	0.5915	−0.2305	0.6071	0.1074	−0.0441	−0.1506
EC	−0.5920	0.2455	−0.5869	0.8303	−0.6345	−0.1499
C	0.6396	−0.3982	0.2871	0.2386	0.3010	−0.2175
Total flavanols	0.5785	−0.2061	0.5515	0.3224	−0.0455	−0.1983
Stilbene						
RES	0.5170	0.2361	0.1400	−0.1428	0.8193	−0.0272
GA	−0.0825	−0.2735	−0.1633	0.4425	−0.0932	−0.1433
VA	0.3331	−0.5280	0.4905	−0.1888	0.2856	0.6684
SI	0.6057	0.1039	0.6802	−0.0965	0.0085	0.3629
PK	−0.3882	0.1884	−0.4302	0.2909	−0.2329	−0.1247
PKEE	0.3370	0.5370	0.0315	0.1148	0.5517	−0.7844
HB	0.2852	0.1187	−0.0561	0.8186	0.6116	0.2844
EL	−0.2802	−0.2311	−0.2089	−0.3980	−0.0622	0.2669
Total DBA	−0.1494	0.2089	−0.4009	0.4818	0.1544	−0.1714
CN	−0.0825	0.5197	−0.0241	0.4609	−0.1805	−0.2683
HCN	0.0363	−0.2673	−0.3495	0.0323	0.2039	0.2007
CA	0.2875	−0.0201	−0.1284	0.2767	0.3739	−0.0385
FER	−0.2594	0.3771	−0.0182	0.0639	−0.0871	−0.8191
CHL	0.4433	0.1137	0.0168	0.2540	0.7850	−0.4495
NCHL	0.1063	0.3430	0.3615	−0.0345	0.0167	−0.7960
CU	0.6144	0.5336	0.4460	0.7101	0.8014	0.5794
SP	0.2539	−0.4558	0.3620	−0.1424	−0.0739	0.2312
Total DCA	0.5427	0.2174	0.1272	0.4031	0.8760	−0.3331

ACW (water-soluble antioxidant capacity), ACL (lipid-soluble antioxidant capacity), TP (total phenolics), TF (total flavonoids), TMAC (total monomeric anthocyanins); Total DBA (total of benzoic acid derivatives), Total-DCA (total of cynnamic acid derivatives); *n* = 32.

## Data Availability

The data presented in this study are available within the article.

## Appendix A

**Table A1:** Monthly temperatures and precipitation in Lednice and Žabčice.

	2014				long-Term			
Month	Annual Temperature [°C]		Sum of Precipitation [mm]		Annual Temperature [°C]		Sum of Precipitation [mm]	
	Lednice	Žabčice	Lednice	Žabčice	Lednice	Žabčice	Lednice	Žabčice
I	1.5	1.1	6.3	22	−1.9	−2	24.3	26
II	3.2	2.7	6.3	12.6	0.3	−0.2	23.9	27
III	8.1	8.5	4	5.6	4.4	4.3	24.8	25
IV	11.6	11.8	20.6	11.2	9.7	9.4	34.7	33
V	14.6	14.5	46.2	62.8	14.5	14.6	57.7	59
VI	18.8	18.8	31.4	43.4	17.5	17.6	66.4	73
VII	21.3	21.5	69.6	85	19.1	19.4	59.8	67
VIII	17.9	17.9	146	113.6	18.4	18.5	50	64
IX	15.4	15.6	166	116.2	14.6	14.8	37.3	38
X	11	11.5	30.1	46.4	9.3	9.3	32.7	38
XI	7.4	7.5	25.2	29.2	4	3.9	41.4	39
XII	2.9	2.4	20.7	28.7	0	0	26.7	30
	11.1 ^a^	11.2 ^a^	572.4 ^a^	576.7 ^a^	9.2 ^a^	9.2 ^a^	479.7 ^a^	519 ^a^
	18.2 ^b^	18.3 ^b^	147.2 ^b^	191.2 ^b^	17 ^b^	17.2 ^b^	184 ^b^	199 ^b^

Superscript ^a^ means the average of annual temperatures and the sum of annual precipitation. Superscript ^b^ means the average temperature and the sum of precipitation during the maturation period from May to July.

**Table A2:** Parameters of calibrations for the used phenolics standards and vitamins.

Compound	Regression Equation ^a^	Regression Coefficient	Linear Range [mg L^−1^]	Retention Time [t_R_ min^−1^]	LOD ^b^ [mg L^−1^]	LOQ ^c^ [mg L^−1^]
RU	y = 0.1264x − 0.0492	*R*^2^ = 0.9998	1.00–100.00	22.84	1.21	3.66
EGC	y = 0.0225x − 0.0272	*R*^2^ = 0.9991	1.00–100.00	10.98	0.05	0.16
EC	y = 0.1382x − 0.1164	*R*^2^ = 0.9992	1.00–100.00	17.21	0.39	1.18
C	y = 0.99x − 0.0176	*R*^2^ = 0.9992	1.00–100.00	12.28	0.22	0.65
QUE	y = 0.3347x − 0.6343	*R*^2^ = 0.9953	5.00–200.00	32.49	5.42	16.43
RES	y = 0.3657x − 0.2952	*R*^2^ = 0.9998	1.00–100.00	27.07	1.23	3.72
GA	y = 0.342x + 0.2116	*R*^2^ = 0.9988	5.00–200.00	2.86	2.77	8.39
VA	y = 0.2604x + 0.0599	*R*^2^ = 1	5.00–200.00	12.67	1.68	5.10
SI	y = 0.3702x + 0.0965	*R*^2^ = 0.9993	1.00–100.00	15.04	0.76	2.31
PK	y = 0.2357x + 0.0111	*R*^2^ = 0.9995	1.00–100.00	5.04	0.41	1.25
PKEE	y = 0.3106x − 0.0751	*R*^2^ = 0.9998	5.00–200.00	25.13	1.13	3.44
HB	y = 0.5091x + 0.1581	*R*^2^ = 0.9996	1.00–100.00	10.03	0.84	2.56
EL	y = 0.2049x − 0.2959	*R*^2^ = 0.9991	5.00–200.00	22.53	2.38	7.20
CN	y = 1.4846x + 0.2387	*R*^2^ = 0.9995	5.00–200.00	24.31	1.78	5.39
HCN	y = 1.263x + 0.6818	*R*^2^ = 0.9989	5.00–200.00	31.01	2.61	7.89
CA	y = 0.5451x + 0.1806	*R*^2^ = 0.9995	1.00–100.00	13.46	0.99	3.00
FER	y = 0.4559x − 0.3865	*R*^2^ = 0.9992	5.00–200.00	20.46	2.26	6.84
CHL	y = 0.2089x + 0.2429	*R*^2^ = 0.9991	1.00–300.00	12.65	0.50	1.53
NCHL	y = 0.1553x + 0.0397	*R*^2^ = 0.9994	0.50–100.00	6.84	0.29	0.89
CU	y = 1.1956x + 0.5853	*R*^2^ = 0.9979	5.00–200.00	18.56	4.39	13.30
SP	y = 0.1757x + 0.0656	*R*^2^ = 0.9994	5.00–200.00	21.51	2.00	6.07
Vitamin C	y = 1.6803x − 16.726	*R*^2^ = 0.9997	5.00–300.00	1.83	3.65	11.05
Vitamin E	y = 0.601x + 0.2111	*R*^2^ = 0.9990	5.00–200.00	4.21	6.95	21.05

^a^ y = peak area and x = concentration (mg L^−1^); ^b^ Limit of detection (S/N = 3); ^c^ Limit of the quantification (S/N = 10). RU (rutin), EGC (epigallocatechin), EC (epicatechin), C (catechin), QUE (quercetin), RES (resveratrol), GA (gallic acid), VA (vanilic acid), SI (syringic acid), PK (protocatechuic acid), PKEE (protocatechuicacid acid ethylester), HB (4-hydroxybenzoic acid), EL (ellagic acid), CN (*t*-cinnamic acid), HCN (hydroxycinnamic acid), CA (caffeic acid), FER (ferulic acid), CHL (chlorogenic acid), NCHL (neochlorogenic acid), CU (*p*-cumaric acid), SP (sinapic acid).
